# Bioinformatics and Molecular Evolution

**DOI:** 10.1002/cfg.486

**Published:** 2005

**Authors:** Damian Counsell

**Affiliations:** MRC Rosalind Franklin Centre for Genomics Research Wellcome Trust Genome Campus Hinxton Cambridge CB10 1SB UK


**Comparative and Functional Genomics**
*Comp Funct Genom* 2005; **6**: 317–319.
Published online in Wiley InterScience (www.interscience.wiley.com). **DOI:** 10.1002/cfg.486

### Book Review

**Bioinformatics and Molecular Evolution.**
By Paul G. Higgs and Teresa K. Attwood. Blackwell: Oxford, UK. ISBN: 1405106832

The field of bioinformatics is still relatively young, but there are already many bioinformatics textbooks for a student to choose from. Writing a good bioinformatics textbook is difficult. Comprehensive study of bioinformatics requires Bachelor's degree-level understanding of at least two kinds: in the molecular/biological and in the computational/mathematical sciences. Both the academic and the practical scope of bioinformatics are incompletely defined and continuously changing. Even when a question is easily described, enduring, and of both academic and commercial importance — for example, the protein folding problem — it is also subtle, messy and (strictly speaking) intractable. As Tim Hubbard, Head of Informatics at the Wellcome Trust Sanger Institute, put it recently when being interviewed about the annotation of new genomes in *The Scientist*: 'We always had this belief that we could automate this problem away, but you can only go so far' (Blackman, 2005). How can practitioners pass on their knowledge of bioinformatics when its substance is so difficult even to pick up and hold?

Despite the challenges, pitfalls and strong competition, it's a credit to the authors of *Bioinformatics and Molecular Evolution*, Paul Higgs and Teresa Attwood, that the worst I can write about it is that its design is unpretty: marred by an unflattering cover, over-dark grey boxouts and a fetish for gradient shading; the book is still usable. The use of boxes, navigational icons, headings and figures is sensible. The layout makes it easy to browse, helps you to find what you want, and leaves hierarchies of ideas and biological systems clear. (As I was writing this review I managed to refer back to passages of interest, even when I had forgotten to note their corresponding page numbers.) The book even includes a helpful flowchart of its own structure. Every chapter begins with a valuable preview of the topics about to be covered and ends with a set of problems and self-test exercises, for which answers are available online, as are figures, data and links. Every chapter ends with a full summary and a set of references. A small thing, I know, but end-of-chapter bibliographies are much more useful than end-of-volume ones and I appreciate the authors' (or editors') choice of the former.

Unlike the exterior, the choice and arrangement of ideas inside the book is tasteful. Many current bioinformatics texts are good users' guides to the confusion of resources available, but this short volume represents a well-rounded programme in classical bioinformatics. It benefits hugely from being based on two university courses (at Manchester in the UK and McMaster in Canada). Its almost-unique selling point is that it sets bioinformatic ideas in the context of evolutionary biology. It is often forgotten by competing works but bioinformatics is, for now at least, just one current in a river of biological thought. Perhaps this is overlooked so often because many bioinformaticians believe that bioinformatics will shift the entire flow of biology.

I intend to enthuse about *Bioinformatics and Molecular Evolution*, but I mention a few of my minor gripes first. One stylistic thing occasionally annoyed me: personal and impersonal pronouns jostle for the foreground: did 'I', 'we' or 'it' write the book? It is refreshing that in their obligatory introduction to basic molecular biology the authors keep bioinformatic principles in mind. It's perhaps less impressive that they criticize current affairs broadcasters for referring to the human genome sequence as 'the genetic code'. 'We have known the genetic code for 40 years!' they exclaim. This minor terminological slip doesn't damage meaning significantly. If they themselves wanted to be truly accurate they should have pointed out that what we have known for 40 years is the *key* to a genetic *cipher*. Every day, agents of the media commit

Copyright © 2005 John Wiley & Sons, Ltd.

crimes against scientific understanding more worthy of the authors' irritation, e.g. the word 'gene' is chronically confused with 'allele'. To their credit, the authors make this distinction prominently and correctly. While I am being pedantic, it's also a shame that the emphasis on molecular genetics didn't prompt a more strict (and a historical) first definition of the term 'homology'.

I also want to quibble slightly with a couple of the broader 'philosophical' positions taken. The chapter on evolution is lucid and informative and covers the neutral theory of mutation generously. This book makes no claim to cover structural bioinformatics extensively, but I can't help feeling that this was a point where protein structure could have been related to sequence in an illuminating way.

Unlike the authors, I don't believe that computer power is now or will be outpaced by biological data; I *do* think that the computing power available to us outpaces our ability to apply it wisely, our ability to think clearly about the biological problems we hope to use it to solve and, indeed, our ability to record our experimental findings meaningfully. For its few tiny faults, this book makes a valuable contribution to tackling these problems.

There are far more things that please me greatly about this book. I will give a few examples that made the strongest impression on me. The authors make one inspired pedagogical connection that I have never seen made explicitly elsewhere: Chapter 2 contains a lean summary of the physicochemical considerations in protein folding, then merges this into a nice introduction to principal components analysis and clustering methods in general. It is also satisfying that this bioinformatics book defines evolution (and indeed life) in some detail and takes the time to dismiss Darwinism's superstitious opponents in a suitably withering way.

The authors' teaching experience shines through in the way they deal with common misconceptions. They are probably used to putting (understandably) confused students right. In addition, for example, to their highlighting of the gene/allele distinction and their tidying up of the bacteria–human horizontal gene transfer story, the authors' treatment of the concept of mitochondrial Eve squishes the common misperception that 'she' was once the only woman on Earth (evolutionary metaphors are powerful and dangerous). Even when the authors are forced to follow the more common approach in practical bioinformatics books of offering an overview of available resources — Chapter 5: 'Information resources for genes and proteins' — it's a relief to find that they do so without resorting to a boring list.

I'm biased but, to me, one of the most important chapters in any textbook of classical bioinformatics has to be the one that covers sequence alignment algorithms — in this case Chapter 6. This progresses gracefully from describing what an algorithm is, through a clear description of big O notation, to explanations of progressively more elaborate dynamic programming approaches. The only apparent miss-step is a slightly ambiguous sentence about implementation of the Smith–Waterman algorithm; elsewhere the difference between local and global and exhaustive and heuristic methods is well delineated. In this chapter and others the authors do their best to connect the underlying theory with its practical consequences for the bioinformatician. So often in current bioinformatics texts these considerations barely meet, although most of the books that cleave them apart are open about their intention to do so.

I am no expert on the wide repertoire of techniques in molecular phylogenetics so I won't pretend to vet the scientific accuracy of Chapter 8, 'Phylogenetic methods', but Higgs and Attwood's explanations lay out the methods clearly and closely enough that a proper overview of the different schools of thought and approaches can be obtained from them.

The easy, conversational and honest prose style of this book is a delight. The authors (and presumably their editors) eschew jargon, portentousness and pseudo-intellectual babble — and bad grammar. One marker of a scientist knowing his or her subject well is an ability to explain its principles in plain English. Chapter 10, 'Probabilistic methods and machine learning', is a perfect example of this. In fewer than 30 pages it explains methods that remain opaque throughout entire volumes dedicated to such subjects and it does so without shying away from the mathematics.

With disarming frankness, the authors begin the one 'bitty' chapter — Chapter 11: 'Further topics in molecular evolution and phylogenetics' — by admitting its bittiness. It would be impossible for Higgs and Attwood to apply their comprehensive but concise summarizing approach as successfully

Copyright © 2005 John Wiley & Sons, Ltd.

to less well-defined and diffuse areas of bioinformatics (this is perhaps one reason why there is less extensive coverage of 'new' bioinformatics — although the book's coverage of 'old' bioinformatics is up to date). I feel as unqualified to judge the science of Chapter 12, 'Genome evolution', as I do the chapter on phylogenetics, but I can only repeat that its contents are also well laid out, seem to review the different approaches used in the area well, and are again easy to read. Chapter 13 'DNA Microarrays and the "omes" ', is devoted to a brisk but well-planned tour of these subjects.

This is not a book I would recommend to those interested in extensive coverage of functional genomics, systems biology or ontologies, but these absences are not disappointments; the emphasis of *Bioinformatics and Molecular Evolution* is clear from its cover — which you wouldn't look behind for coverage of structural bioinformatics either. While the last is a more long-established field of study, there are, however, several very good structural bioinformatics textbooks already on the market. Coming this 'late' to the game, the authors have been wise to play to their strengths and produce a book that is both distinctive and reasonably wide-ranging.

Rigorous as the book is, neither would you turn to it for a proof that a particular problem in bioinformatics is NP-complete. And a good thing too. I am a pragmatic bioinformatician, happy to recommend any bioinformatics textbook that is accurate, brief and economical and begins its discussion of alignment statistics with the heading 'Why bother with statistics?' — before going on to bother with the relevant statistics efficiently and thoughtfully.

Although it is not a 'how to'-style practical text, it is full of sensible practical advice. It acknowledges both the biological and mathematical underpinnings of the subject. It does not cover all aspects of bioinformatics, but does not pretend to. What it does cover it handles elegantly. *Bioinformatics and Molecular Evolution* is an excellent and current introduction to classical bioinformatics and an ideal core text for a Master's course in the subject.

### Reference

Blackman S. 2005. The hum and the genome. *Scientist* **19**: 15.

Damian Counsell

*MRC Rosalind Franklin Centre for Genomics Research, Wellcome Trust Genome Campus, Hinxton, Cambridge CB10 1SB, UK.*
*E-mail: bioinformatics@counsell.com*
*Web:* ***http://www.counsell.com/academic/***

Copyright © 2005 John Wiley & Sons, Ltd.

